# Paratracheal Cysts

**DOI:** 10.5811/cpcem.2017.2.33297

**Published:** 2017-07-06

**Authors:** Mohammad R. Mohebbi, Chadd K. Kraus

**Affiliations:** * University of Missouri-Columbia, Department of Emergency Medicine, Columbia, Missouri; † Geisinger Health System, Department of Emergency Medicine, Danville, Pennsylvania

## CASE PRESENTATION

**Image 1:**
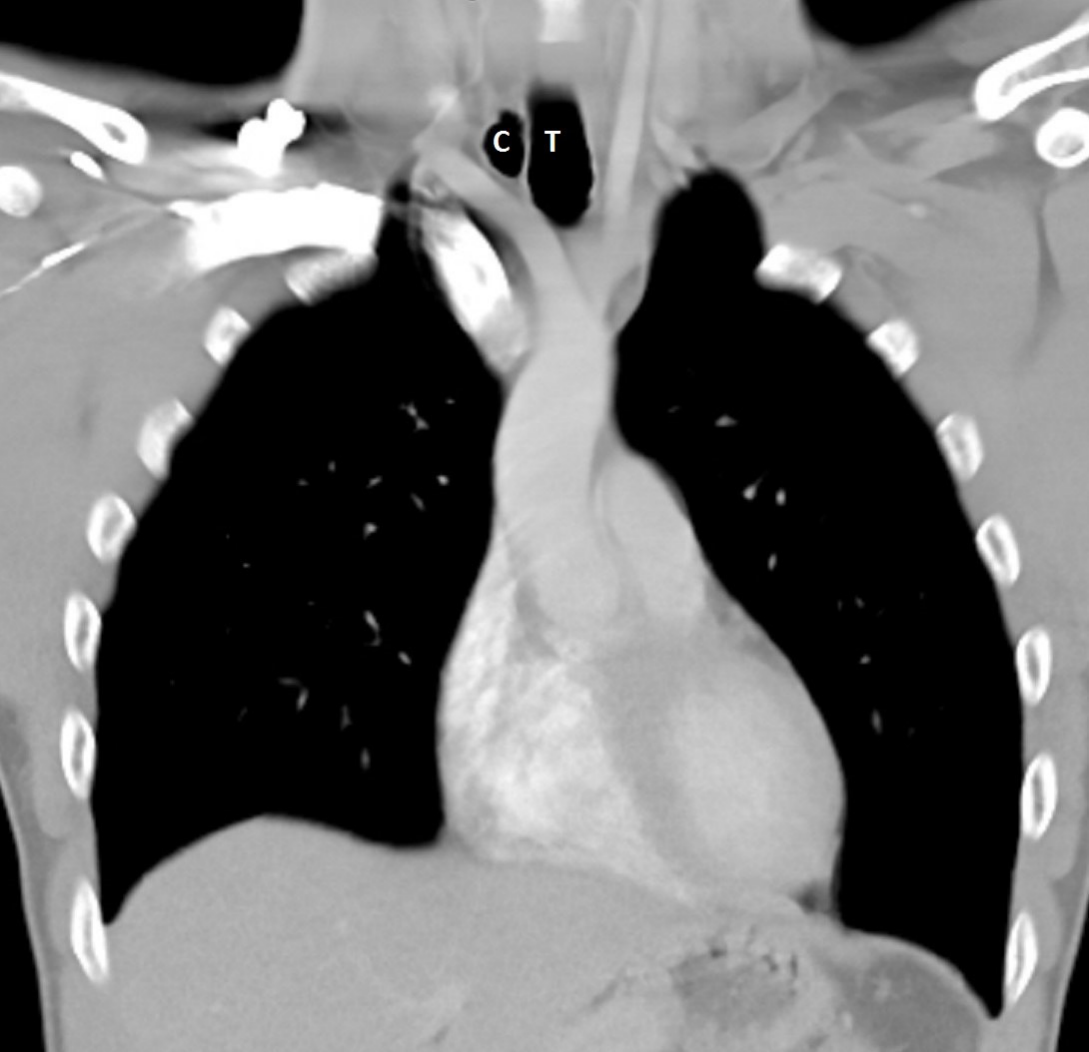
Right-sided paratracheal cyst in the thoracic outlet. *T*, trachea; *C*, paratracheal cyst.

**Image 2:**
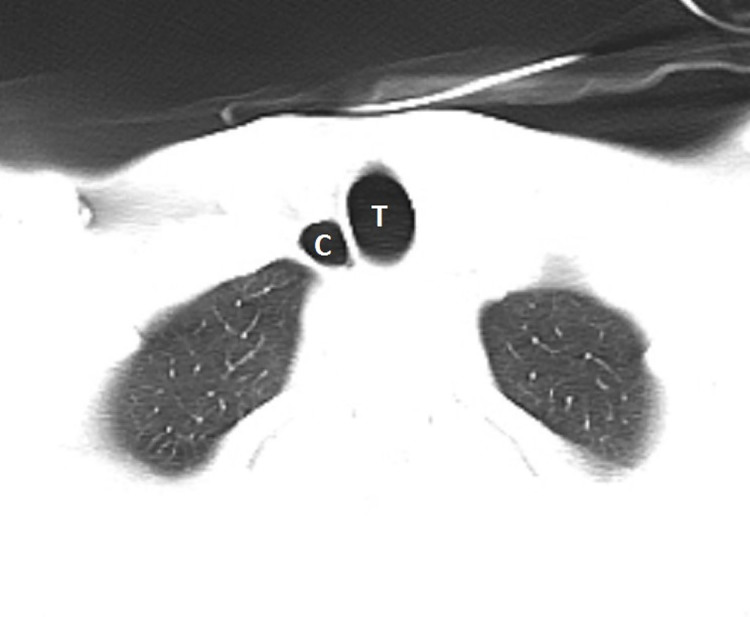
No evidence of lung tissue in the air collection in the lung window image. *T*, trachea; *C*, paratracheal cyst.

A 38-year-old male with no significant past medical history presented to the emergency department with pain and swelling on the left mandibular area and the right upper quadrant of the abdomen after a reported assault in which he was punched in the face and kicked in the right chest wall during a fight at a bar. The patient reported that he had been drinking alcohol but denied loss of consciousness. Vital signs were within normal limits and physical exam was otherwise unremarkable. Due to the patient’s history and intoxication, computed tomography (CT) of the head, cervical spine, chest and abdomen were obtained and were unremarkable with the exception of a collection of air on the right side of the upper trachea and anterior to the right lung apex (Image 1). It did not seem to communicate with either the lung or trachea and there was no evidence of lung tissue in the air collection in the lung window images (Image 2). However, the patient had reported trauma to the right chest/abdominal wall. After consulting the otolaryngology and pulmonology services, a conclusion was made that this was an incidental finding of paratracheal cyst and unrelated to the trauma reported by the patient.

## DISCUSSION

Right paratracheal cysts are a common finding on CT and are reported in just under 4% of the United States population.^1^ In the setting of trauma, paratracheal cysts can mimic pneumomediastinum. The nature of the paratracheal cyst is diverse; however, they could be considered to be tracheal diverticula,^2^ although a tract to the trachea or tracheal mucosal is not always found on broncoscopy. There is a potential association of paratracheal cysts with obstructive lung disease^1^,^2^ and chronic cough,^3^ although the cysts have been found in pediatric patients as well.^1^,^4^ Paratracheal cysts are usually an incidental finding on CT imaging. It is important for emergency physicians to be aware of this entity and be mindful of this benign finding, especially in the setting of trauma, where it could be confused with an injury depending on the history, mechanism of injury and clinical exam The anatomic location of the cysts could also complicate certain procedures such as central venous catheterization of the internal jugular vein or make these procedures more difficult.

CPC-EM Capsule

What do we already know about this clinical entity?

Paratracheal cysts are a common finding on CT. They can mimic pneumomediastinum. There is a potential association with chronic obstructive pulmonary disease, and chronic cough. The cysts have also been reported in children.

What is the major impact of the image(s)?

This incidental benign finding can be a diagnostic dilemma in the setting of trauma and could be confused with an acute injury.

How might this improve emergency medicine practice?

Emergency physicians should be mindful of this benign finding, especially in the setting of trauma. The anatomic location of the cysts may also complicate procedures such as internal jugular line placement.
